# CUEDC2 controls osteoblast differentiation and bone formation via SOCS3–STAT3 pathway

**DOI:** 10.1038/s41419-020-2562-5

**Published:** 2020-05-11

**Authors:** Jung-Woo Kim, Sin-Hye Oh, Mi Nam Lee, Ju Han Song, Byung-Chul Jeong, Jin-Woo Yang, Xianyu Piao, Yaran Zang, Je-Hwang Ryu, Jeong-Tae Koh

**Affiliations:** 1 Hard-Tissue Biointerface Research Center, School of Dentistry, Chonnam National University, Gwangju, 61186 Republic of Korea; 2 Department of Pharmacology and Dental Therapeutics, School of Dentistry, Chonnam National University, Gwangju, 61186 Republic of Korea; 3 Central R&D Center, KOFEC, Gokseong, Jeollanamdo, 57525 Republic of Korea

**Keywords:** Bone development, Differentiation

## Abstract

The CUE domain-containing 2 (CUEDC2) protein plays critical roles in many biological processes, such as the cell cycle, inflammation, and tumorigenesis. However, whether CUEDC2 is involved in osteoblast differentiation and plays a role in bone regeneration remains unknown. This study investigated the role of CUEDC2 in osteogenesis and its underlying molecular mechanisms. We found that CUEDC2 is expressed in bone tissues. The expression of CUEDC2 decreased during bone development and BMP2-induced osteoblast differentiation. The overexpression of CUEDC2 suppressed the osteogenic differentiation of precursor cells, while the knockdown of CUEDC2 showed the opposite effect. In vivo studies showed that the overexpression of CUEDC2 decreased bone parameters (bone volume, bone area, and bone mineral density) during ectopic bone formation, whereas its knockdown increased bone volume and the reconstruction percentage of critical-size calvarial defects. We found that CUEDC2 affects STAT3 activation by regulating SOCS3 protein stability. Treatment with a chemical inhibitor of STAT3 abolished the promoting effect of CUEDC2 silencing on osteoblast differentiation. Together, we suggest that CUEDC2 functions as a key regulator of osteoblast differentiation and bone formation by targeting the SOCS3–STAT3 pathway. CUEDC2 manipulation could serve as a therapeutic strategy for controlling bone disease and regeneration.

## Introduction

Bone is a hard tissue that forms the skeleton and regulates and maintains the concentration of ions such as calcium and phosphate^1^. The bone homeostasis is regulated by maintaining a balance between osteoblasts and osteoclasts^2^. Differentiation of osteoblasts is initiated by cytokines such as bone morphogenetic protein 2 (BMP2), and the differentiation process is regulated not only by various transcription factors that control osteogenic gene expression but also by the adapter proteins^1^. Recently, ubiquitination-associated adapter proteins have been proposed as important coordinators of signaling pathways in osteoblast differentiation^3,4^.

CUE domain-containing 2 (CUEDC2) protein is an adapter protein that contains a ubiquitin-binding motif within a CUE domain. This domain plays a dual role not only in monoubiquitin and polyubiquitin interactions but also in regulating protein stability via ubiquitination of specific substrates^5^. CUEDC2 is expressed in various tissues and organs including heart, and regulates diverse cellular functions such as the cell cycle, steroid receptor modulation, inflammatory reactions, and intracellular reactive oxygen production^6–10^. Although evidence for the multifunctional properties of CUEDC2 is accumulating, to date, there are no studies of the role of CUEDC2 in cell differentiation.

Recent studies have shown that CUEDC2 negatively regulates the phosphorylation of signal transducer and activator transcription factor 3 (STAT3) by enhancing the stability of suppressor of cytokine signaling 1 (SOCS1) or SOCS3 proteins, which regulate tumorigenesis^11,12^. STAT3 is a transcription factor that is activated by phosphorylation of its tyrosine 705 and serine 727 residues in response to various cytokines (e.g., LIF, EGF, IL-6, OSM, and BMP2)^13–17^. Phosphorylation of STAT3 is considered an essential factor for osteoblast differentiation in human mesenchymal stem cells, which are the progenitors of osteoblasts^18,19^. In osteoblasts and vascular smooth muscle cells, phosphorylated STAT3 promotes RUNX2 expression by directly binding to the promoter region of RUNX2^20,21^. Osteoblast or osteocyte-specific STAT3 deficiency reduces weight and bone mass and causes spinal malformations^22–24^, suggesting an essential role for STAT3 in bone development.

Considering the role of STAT3 in osteoblast differentiation and the relationship between STAT3 and CUEDC2, we questioned whether CUEDC2 plays a specific role in bone tissue. In this study, we found that CUEDC2 directly controls osteoblast differentiation via regulating SOCS3-mediated STAT3 activation and further demonstrated the osteogenic role of CUEDC2 in an in vivo bone-forming model. Our findings suggest a potential role for CUEDC2 as a novel target in treatments involving bone regeneration.

## Materials and methods

### Antibodies and chemicals

Antibodies against CUEDC2 (#79036) and OSX (#22552) were purchased from Abcam (Cambridge, UK) and antibodies against Flag-tag (#2368), RUNX2 (#8466), p-STAT3 (#4113), and MEK2 (#9125) were purchased from Cell Signaling Technology (Danvers, MA, USA). β-ACTIN (#47778), UB (#47721), and LAMIN B (#56145) antibodies were obtained from Santa Cruz Biotechnology (Dallas, TX, USA). The SOCS1 (#PA1-29533) and SOCS3 (#PA1-29534) antibodies were purchased from Thermo Fisher Scientific (Waltham, MA, USA). HA-tag (#H9658) antibody and Stattic were purchased from Sigma-Aldrich (St. Louis, MO, USA).

### Osteoblast differentiation and adenoviral infection

Primary mouse bone marrow mesenchymal stem cells (BMSCs) were isolated from the tibias and femurs of 6-week-old C57BL/6 male mice. The cells were cultured in α-minimal essential medium (α-MEM, Gibco) supplemented with 1% penicillin/streptomycin (P/S, Gibco), and 10% fetal bovine serum (FBS, Gibco). After 3 days, cell adhesion and population were monitored, and the medium exchanged for freshness. In this study, BMSCs from passage 2–3 were used. MC3T3-E1 cells were cultured under the same conditions as BMSCs. To induce osteogenic differentiation, BMSCs and MC3T3-E1 cells were cultured as previously described conditions^25^. For viral infection, the cells were treated with adeno-associated viruses containing the CUEDC2 (Ad-CUEDC2, Vector Biolabs, Malvern, PA, USA) or CUEDC2-specific short hairpin RNA (Ad-shCUEDC2, Vector Biolabs) or green fluorescence protein (Ad-GFP, Vector Biolabs) with the indicated viruses at the designated multiplicity of infection in serum-free medium. After 6 h of incubation with the viruses, the medium was exchanged with media containing 10% FBS and the infected cells cultured in humidified air containing 5% CO_2_ at 37 °C for 24 h.

### Osteoclast differentiation and retroviral infection

Primary mouse bone marrow cells were isolated from the tibia and femur of 6-week-old male C57BL/6 mice. Bone marrow cells were cultured in α-MEM supplemented with 1% P/S and 10% FBS in the presence of 30 ng/ml M-CSF (Biolegend, San Diego, CA, USA). After 3 days, bone marrow-derived monocyte/macrophage cells (BMMs) were cultured with M-CSF and RANKL (Peprotech, Rocky Hill, NJ, USA) to induce osteoclast differentiation for 3 days. For retroviral infection, pMX-IRES-EGFP (pMX-GFP) or pMX-IRES-CUEDC2 (pMX-CUEDC2) were transfected into Plat-E cells using FuGENE 6 (Promega, Madison, WI, USA). After 48 h, BMM cells were cultured with preconditioned medium from Plat-E cells for 6 h with 10 µg/ml polybrene (Sigma-Aldrich).

### Transient transfection and plasmids

Transient transfections of mammal expression plasmids and siRNA were performed with Lipofectamine 2000 or Lipofectamine RNAiMAX (Invitrogen) as described previously^25^. Flag and Myc-CUEDC2 expression vectors were purchased from Origene (Rockville, MD, USA). Plasmids for HA-SOCS3 and HA-UB were constructed in a CMV promoter-derived expression vector. Predesigned and validated CUEDC2 (1344394) siRNA and Si-Control nontargeting siRNA were purchased from Bioneer (Daejeon, South Korea). The total amounts of transfected plasmids were equalized across the groups by the addition of an empty vector.

### RNA isolation and RT-PCR analysis

Total RNA was isolated from cultured cells using TRIzol reagent (Invitrogen). cDNA was synthesized using random primers, MMLV, dNTP, and RNAsin (Promega) according to the manufacturer’s instructions. For quantitative analysis, real-time PCR was performed using the StepOnePlus^TM^ real-time PCR system (Thermo Fisher Scientific) and Power SYBR green PCR master mix (Thermo Fisher Scientific). PCR was performed at 95 °C for 5 min followed by 40 cycles of 95 °C for 30 s, 55 °C for 30 s, and 72 °C for 30 s. Quantitative analysis was performed using StepOne Software v2.1 (Thermo Fisher Scientific). Relative target gene expression was quantified using the comparative Ct method. The sequences of primers used for real-time PCR are shown in Supplementary Table 1.

### Immunoprecipitation and western blotting

Total cell extract was prepared using lysis buffer (Cell Signaling Technology) and centrifuged at 16,000 × *g* for 15 min at 4 °C. For immunoprecipitation, HEK293T cells were transiently co-transfected with HA-SOCS3, Flag-CUEDC2, and HA-UB. Cell lysates were precleared with protein G-agarose beads (Invitrogen) and were then incubated with the indicated antibodies overnight at 4 °C. After incubation with protein G-agarose beads for 2 h, the suspension was centrifuged, and the beads were washed with lysis buffer three times. The immunoprecipitated materials were solubilized in SDS sample buffer (Sigma-Aldrich). Total proteins or immunoprecipitated proteins were resolved on a SDS-PAGE gel and transferred into a PVDF membrane. After blocking with 5% milk in Tris-buffered saline containing 0.1% Tween-20, the membrane was incubated with specific primary antibodies. Signals were visualized using an enhanced chemiluminescence reagent (Millipore, Billerica, MA, USA) in a LAS-4000 Lumino Image Analyzer System (Fujifilm, Tokyo, Japan). For quantitative analysis, the blotting results were quantified using Multi Gauge V3.0 software (Fujifilm).

### Alkaline phosphatase (ALP) staining and alizarin red (AR) staining

To evaluate ALP enzyme activity and mineral deposition, ALP staining and AR staining were performed as described previously^25^. For quantitative analysis, the staining in ALP-positive cells was measured using Image J software (National Institutes of Health, Bethesda, MD, USA). The AR stains were extracted using 10% cetylpyridinium chloride prepared in 10 mM sodium phosphate solution (pH 7.0). The absorbance was then measured at a wavelength of 540 nm on a spectrophotometer (Thermo Fisher Scientific).

### TRAP staining

To observe osteoclast activity, cultured cells were fixed with 10% formaldehyde for 15 min, and then TRAP staining was performed using a TRAP stain kit (Cosmo Bio, Tokyo, Japan) according to the manufacturer’s instructions. After washing with distilled water, stained cells were observed by optical microscopy (Leica Microsystems, Wetzlar, Germany). For quantitative analysis, TRAP-positive multinucleated cells (MNCs, *n* > 3) were counted.

### Ectopic and orthotopic bone formation models

All animal experiments were performed according to the guidelines of the Chonnam National University Animal Care and Use Committee (CNU IACUC-YB-2017-73). C57BL/6 mice were purchased from Damool Science (Daejeon, Korea). Six-week-old male mice were randomly assigned to each experimental group. The mice were anesthetized by intraperitoneal injection of Zoletil (30 mg/kg; Virbac, Carros Cedex, France) and Rompun (10 mg/kg; Bayer Korea, Seoul, Korea). For the ectopic bone formation model, the dorsal skins of anesthetized mice were implanted with collagen sponges containing Ad-GFP or Ad-CUEDC2 with BMP2 subcutaneously (*n* = 3). For the orthotopic bone formation model, the scalps of mice were dissected, and the periosteum was removed. The exposed calvarial bone was drilled to create a 5-mm opening using a trephine bur (Fine Science Tools, Foster City, CA, USA). Absorbable collagen sponges with Ad-GFP or Ad-shCUEDC2 mixed in the presence of PBS or BMP2 were implanted in the bone defect site (*n* = 3). After 4 weeks, all mice were sacrificed with CO_2_ to evaluate bone regeneration.

### Micro-computed tomography (µ-CT) scanning and analysis

Specimens for ectopic and orthotopic bone formation were scanned by µ-CT (Skyscan 1172; Bruker, Kontich, Belgium). Scan conditions were performed as described previously^25^. Three-dimensional images were obtained using Mimics software (Materialise, Leuven, Belgium) and bone parameters were analyzed using the CT analyzer program (Bruker).

### Immunohistochemistry

Immunohistochemistry (IHC) analysis was performed using the VECTASTAIN ABC kit (Vector Laboratories, Burlingame, CA, USA) according to the manufacturer’s protocol. Briefly, the tissues were deparaffinized in xylene and rehydrated in ethanol. To inhibit peroxidase activity, the tissues were incubated in hydrogen peroxide for 30 min at room temperature and treated with normal horse serum for 1 h to suppress nonspecific reactions. They were then incubated with specific primary antibody (CUEDC2, 1:100) or normal rabbit IgG overnight. The tissues were incubated with biotin-labeled secondary antibody for 1 h and developed with a DAB substrate kit (Vector Laboratories). Then, hematoxylin was used as counterstains. IHC images were obtained using an optical microscopy (Leica Microsystems).

### Statistical analysis

Statistical analysis of the data was performed using Student’s *t* test or analysis of variance with Tukey’s multiple comparison test on Prism5 software (GraphPad Software, San Diego, CA, USA). Differences were considered significant at *P* < 0.05. The results are presented as the mean ± standard deviation of triplicate samples. All experiments were repeated at least three times.

## Results

### Expression pattern of CUEDC2 during osteogenesis

**Fig. 1:**
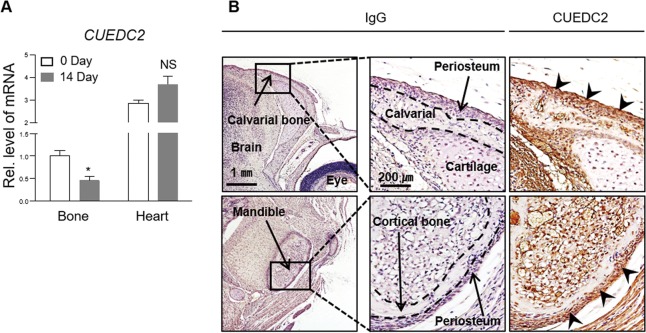
Decreased CUEDC2 expression is correlated with bone development. **a** The expression levels of CUEDC2 mRNA in calvarial bone and heart tissues of mice at postnatal days 0 and 14 were evaluated by real-time PCR. CUEDC2 levels in calvarial bone at day 0 were normalized to 1, and heart tissue was used as a positive control. *P < 0.05 versus day 0. NS, nonsignificant. **b** The expression of CUEDC2 protein in skull tissues of mice at postnatal day 0 was observed using immunohistochemistry. The broken line of the middle panel indicates the bone boundary. The arrowhead in the right panel indicates the positive staining of the CUEDC2 protein in the periosteum.

To identify the expression of CUEDC2 in bone tissue, we first analyzed CUEDC2 mRNA levels during the development of calvarial bone tissue, using heart tissues as a positive control^9^. RT-PCR analysis showed that CUEDC2 mRNA expression during bone development was significantly decreased by about 50% (Fig. 1a). Further, when IHC was performed on the entire skull of newborn mice, CUEDC2 was strongly expressed in the periosteum, where many undifferentiated cells are present, whereas it was weakly expressed in highly differentiated osteocytes (Fig. 1b).

**Fig. 2:**
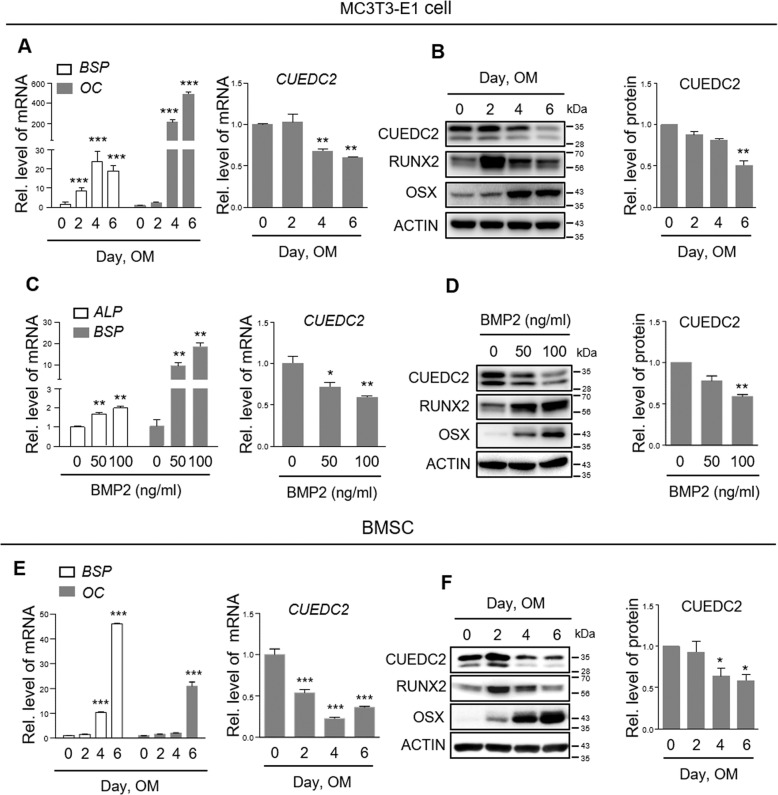
CUEDC2 is downregulated during osteoblast differentiation. **a**, **b**, **e**, **f** MC3T3-E1 cells and BMSCs were cultured in osteogenic medium [OM; ascorbic acid (50 µg/ml), β-glycerophosphate (5 mM), BMP2 (50 ng/ml)] for 6 days. **c**, **d** MC3T3-E1 cells were cultured with BMP2 (50, 100 ng/ml) for 2 days. **a**, **c**, **e** The expression of BSP, OC, and CUEDC2 mRNA was evaluated by real-time PCR using specific primers. *P < 0.05, **P < 0.01, ***P < 0.001 versus day 0 or BMP2 0 ng/ml. **b**, **d**, **f** The levels of RUNX2, OSX, and CUEDC2 proteins were evaluated by western blotting using specific antibodies. Right panels are a quantification of the CUEDC2 bands by densitometry using Science Lab Image Gauge version 3.0 software (Fujifilm). *P < 0.05, **P < 0.01 versus day 0 or BMP2 0 ng/ml.

Next, we tested the expression of CUEDC2 during BMP2-induced osteoblast differentiation in MC3T3-E1 cells and primary BMSCs. During the osteoblast differentiation process induced by osteogenic medium containing BMP2, the levels of RUNX2, OSX, ALP, BSP, and OC, which are typical osteoblast differentiation markers, were significantly increased in MC3T3-E1 cells and BMSCs (Fig. 2a, b, e, f), consistent with our previous reports^26,27^. Interestingly, the mRNA and protein levels of CUEDC2 decreased significantly in a time-dependent (Fig. 2a, b, e, f) and BMP2 dose-dependent manner (Fig. 2c, d). These results imply that osteogenic stimulation could reduce the level of CUEDC2 and may be involved in osteoblast differentiation and bone development.

### CUEDC2 negatively regulates osteogenesis

**Fig. 3:**
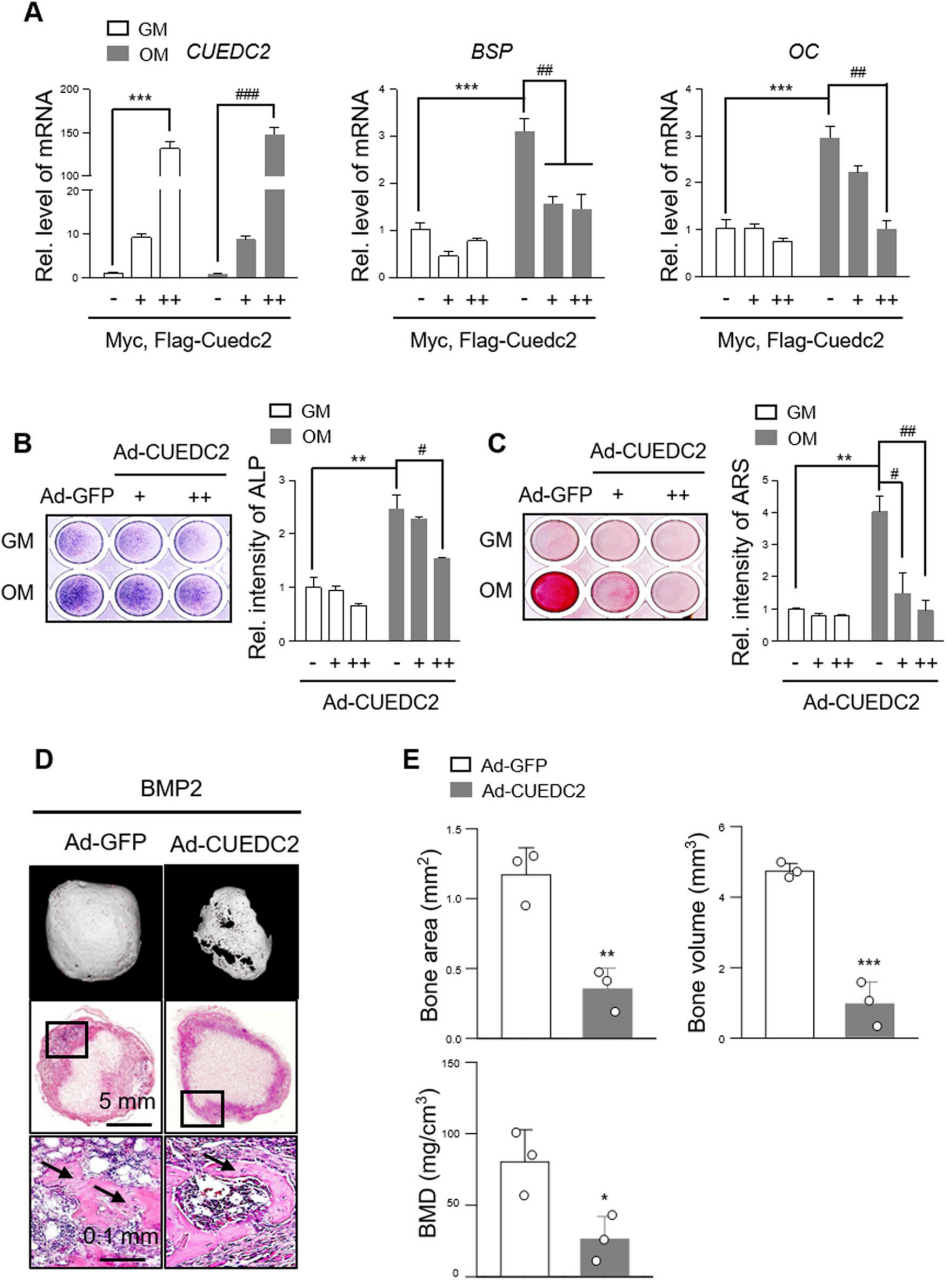
Overexpression of CUEDC2 inhibits osteogenesis in vitro and in vivo. **a** MC3T3-E1 cells were transfected with Myc-Flag tagged CUEDC2 (+, 500 ng; ++, 1000 ng) or an empty vector and cultured for 4 days in GM (growth medium) or OM (osteogenic medium). Real-time PCR was performed with CUEDC2-, BSP-, and OC-specific primers. ***P < 0.001 versus the GM control group. ^##^*P* < 0.01, ^###^*P* < 0.001 versus control of the OM group. **b**, **c** MC3T3-E1 cells infected with Ad (adenovirus)-GFP (100 MOI) or Ad-CUEDC2 (+, 50 MOI; ++, 100 MOI). **b** After infection, the cells were cultured with GM or OM for 5 days and stained with BCIP/NBT solution for alkaline phosphatase (ALP) staining. For quantitative analysis, the intensity of ALP staining was measured using Image J software. **c** Infected cells were cultured for 12 days under the conditions shown in the Fig. 3b legend. Alizarin red (AR) staining. For quantitative analysis of AR staining, the stained cells were extracted with 10% cetylpyridinium chloride and measured at 562 nm using a multiplate reader. **P < 0.01 versus Ad-GFP of the GM group. ^#^*P* < 0.05, ^##^*P* < 0.01 versus Ad-GFP of the OM group. **d**, **e** Ad-GFP (1 × 10^10^ virus particles) or Ad-CUEDC2 (1 × 10^10^ virus particles) with BMP2 (3 µg) were implanted in a subcutaneous pocket on the backs of mice using an absorbable collagen sponge (*n* = 3). After 4 weeks, mice were sacrificed and evaluated radiologically or histologically. **d** Top panels display 3D images of regenerated ectopic bone. Bottom panels display the inside of the bone with hematoxylin and eosin staining. The arrow indicates newly regenerated bones. **e** The area, volume, and mineral density of regenerated bone was measured using the CTAn program. *P < 0.05, **P < 0.01, ***P < 0.001 versus Ad-GFP. BMD bone mineral density.

To examine the functional role of CUEDC2 in osteoblast differentiation, CUEDC2 was overexpressed in MC3T3-E1 cells and BMSCs using CUEDC2 vector or Ad-CUEDC2. In both overexpression methods for CUEDC2, the protein level was measured using western blotting (Fig. S1a, b). When CUEDC2 was overexpressed in MC3T3-E1 cells, the levels of BSP and OC mRNA were significantly decreased (Fig. 3a). In addition, the overexpression of Cuecd2 using Ad-CUEDC2 infection significantly inhibited BMP2-induced ALP activity (Fig. 3b) and mineralized nodule formation (Fig. 3c). These results were consistent with those of the experiments using BMSCs (Fig. S2a, c, d).

Next, we assessed the role of CUEDC2 in BMP2-induced ectopic bone formation in vivo. When a collagen sponge containing Ad-GFP with BMP2 was dorsally implanted in a subcutaneous pocket, ectopic bone formation was induced by BMP2, whereas the implantation of a collagen sponge containing Ad-CUEDC2 with BMP2 significantly reduced the ectopic bone formation (Fig. 3d). A quantitative µ-CT analysis also confirmed that the overexpression of CUEDC2 significantly decreased BMP2-induced bone parameters such as bone area, bone volume, and bone mineral density (Fig. 3e). Histological analysis showed that BMP2 regenerated new cortical like bone as well as the trabecular like bone, whereas the overexpression of CUEDC2 resulted in sparsely cortical like bone formation without trabecular like bone (Fig. 3d). These results suggest that CUEDC2 negatively regulates the maturation of osteoblast cells and further inhibits bone formation.

### Suppression of CUEDC2 provides an opportunity to improve osteogenesis

**Fig. 4:**
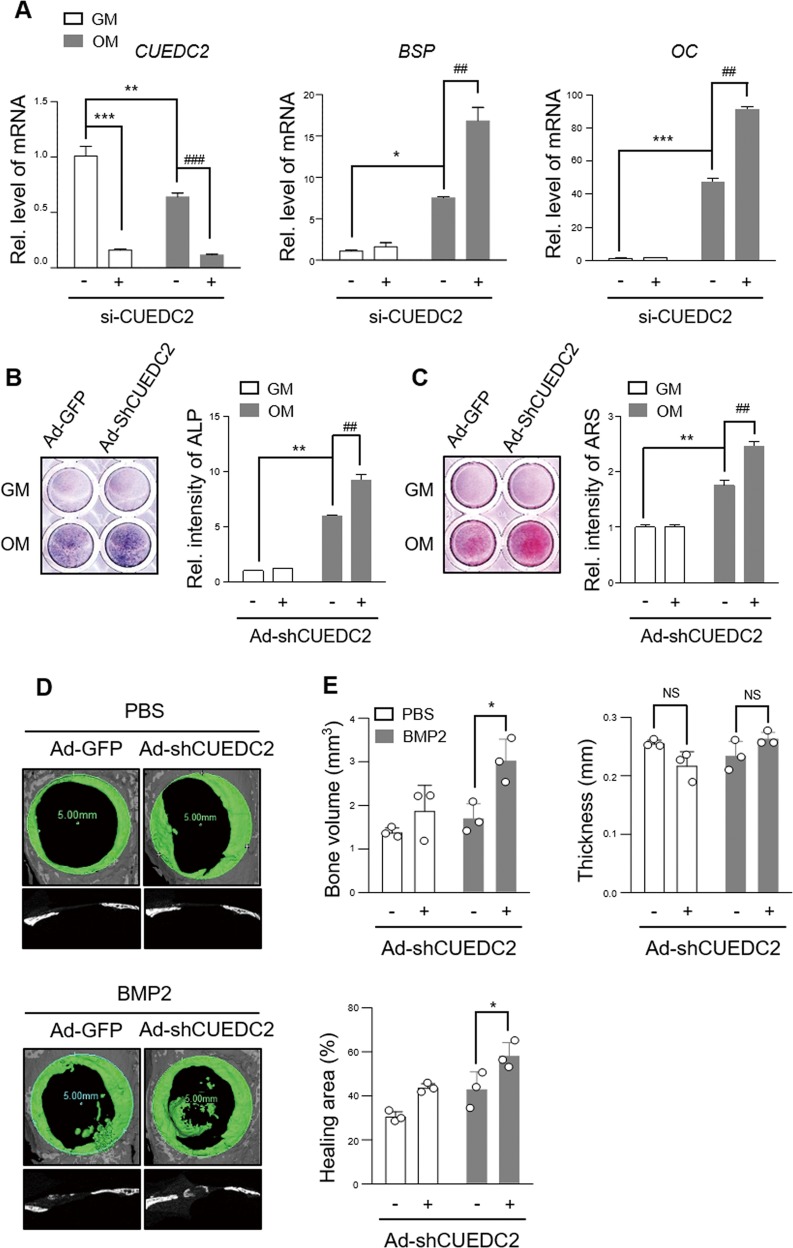
Suppression of CUEDC2 accelerates osteogenesis in vitro and in vivo. **a** MC3T3-E1 cells were transfected with siRNA CUEDC2 (25 nM) or siRNA control (25 nM). After 24 h, cells were cultured with GM or OM for 4 days. Real-time PCR analysis was performed using BSP-, OC-, and CUEDC2-specific primers. *P < 0.05, **P < 0.01, ***P < 0.001 versus si-control of the GM group. ^##^*P* < 0.01, ^###^*P* < 0.001 versus the OM group si-control. **b**, **c** MC3T3-E1 cells were infected with Ad-GFP (50 MOI) or Ad-shRNA CUEDC2 (50 MOI) for 24 h. The infected cells were cultured under the same conditions as those mentioned in the legend of Fig. 3b, c. **b** ALP staining. **c** AR staining. **P < 0.01 versus Ad-GFP of the GM group. ^##^*P* < 0.01 versus Ad-GFP of the OM group. **d**, **e** A critical-sized bone defect with a diameter of 5 mm was created using a trephine bur. Ad-GFP (7 × 10^9^ virus particle) or Ad-ShCUEDC2 (7 × 10^9^ virus particles) with PBS or BMP2 (500 ng) were implanted in punched calvarial bones of mice using an absorbable collagen sponge (*n* = 3). After 4 weeks, the calvarial bone was harvested and examined using micro-CT. **d** The panels on top display 3D images of regenerative bone (green). Bottom panels display the sagittal view of the calvarial bone. **e** The volume, thickness, and healing area of regenerated bone were measured using the CTAn program. *P < 0.05 versus Ad-GFP with the BMP2 group.

The preceding results indicated that CUEDC2 plays a negative role in osteoblast differentiation and bone formation. Therefore, we explored the therapeutic potential of CUEDC2 knockdown. For evaluating the efficiency of si-CUEDC2 or Ad-shCUEDC2, we analyzed the protein levels by western blotting (Fig. S1c, d). When CUEDC2 was silenced in MC3T3-E1 cells, the levels of BSP and OC mRNA significantly increased by approximately twofold compared with those in the BMP2 group (Fig. 4a). In addition, the lack of CUEDC2 enhanced ALP activity (Fig. 4b) and mineralized nodule formation (Fig. 4c). These results are consistent with those of experiments using BMSCs (Fig. S2b, c, d).

To further investigate the therapeutic potential of CUEDC2 suppression on bone defects, we created critical-sized bone defects in mouse calvaria, and then implanted a collagen sponge into the defect region, as shown in Fig. 4d. Three-dimensional image analysis showed that the implantation of BMP2 with Ad-shCUEDC2 accelerated bone formation (Fig. 4d). The quantitative μ-CT analysis also showed that the bone volume and healing area significantly increased in the Ad-shCUEDC2-containing BMP2 group compared with those in the Ad-GFP-containing BMP2 group (Fig. 4e). However, the thickness of newly formed bone showed no difference (Fig. 4e). These results demonstrate that suppression of CUEDC2 has therapeutic potential for the restoration of bone loss.

### CUEDC2 controls STAT3-mediated osteogenesis by regulating SOCS3 protein stability

**Fig. 5:**
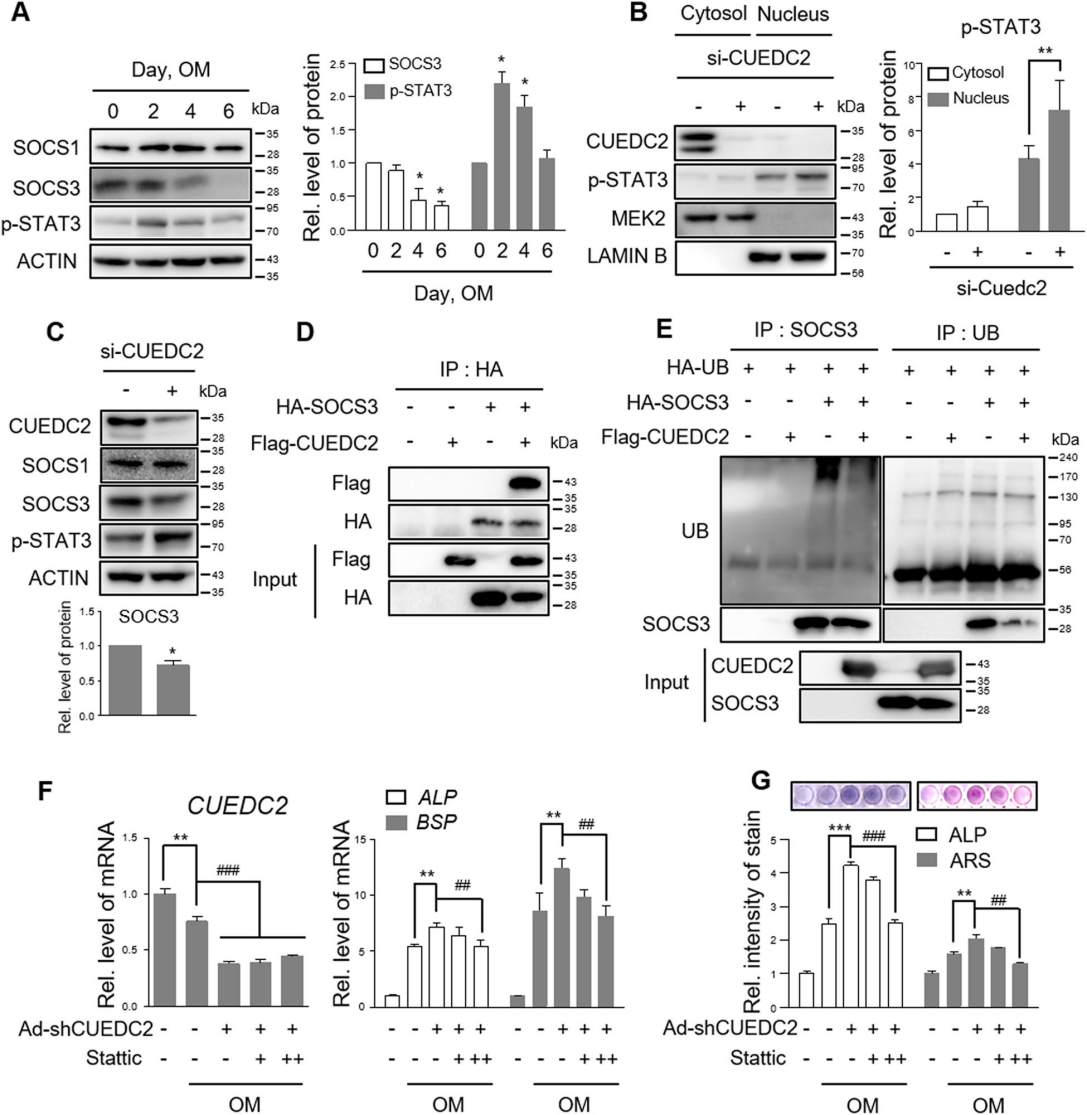
CUEDC2 inhibits STAT3-mediated osteogenesis by regulating SOCS3 protein stability. **a**–**c** The level of SOCS1, SOCS3, and phosphorylated STAT3 proteins was evaluated by western blotting. The relative protein levels of the indicated proteins were calculated after normalization to β-ACTIN. *P < 0.05, **P < 0.01 versus control. **a** MC3T3-E1 cells were cultured for 6 days under the conditions shown in the Fig. 2b legend. **b**, **c** MC3T3-E1 cells transfected under the same conditions as the knockdown (Fig. 4a) of CUEDC2. After 24 h, cells were cultured with GM for 1 day. **b** The cytosolic and nuclear proteins were extracted from the cells for western blotting. MEK2 and LAMIN B were used as a loading control. The interaction between CUEDC2 and SOCS3 proteins (**d**) and the level of ubiquitin**-**conjugated SOCS3 proteins (**e**) were evaluated. HEK293T cells were co**-**transfected with indicated plasmids, as shown in **d**, **e**. Cell lysates were immunoprecipitated with anti-HA (**d**) or anti-SOCS3 or anti-UB (**e**) followed by western blotting with the indicated antibodies. As an input, 1% cell lysates were loaded. **f**, **g** Infection with Ad-shCUEDC2 or Ad-GFP was performed under the same conditions as described in the legend of Fig. 3b. After infection, the cells were cultured with Stattic (+, 2.5 µM; ++, 5 µM) in the presence of OM to investigate osteoblast marker gene expression (2 days), ALP activity (5 days), and mineralized nodule formation (12 days). **P < 0.01, ***P < 0.001 versus OM. ^##^*P* < 0.01, ^###^*P* < 0.001 versus Ad-shCUEDC2 with OM. **f** Real-time PCR analysis. **g** ALP and AR staining.

CUEDC2 regulates the phosphorylation of STAT3 by regulating the stabilization of the SOCS1 or SOCS3 protein in tumor cells^11,12^. Therefore, we measured changes in the levels of SOCS1, SOCS3, and phosphorylated STAT3 after BMP2 treatment or CUEDC2 silencing in MC3T3-E1 cells. STAT3 phosphorylation was increased by BMP2 and reached its peak on day 2 (Fig. 5a). The knockdown of CUEDC2 increased the phosphorylation of STAT3 in the cytosol, nucleus, and whole lysates (Fig. 5b, c). In contrast, SOCS3 was significantly decreased during osteoblast differentiation and in CUEDC2-silenced cells (Fig. 5a, c). There was no change in the SOCS1 protein level despite CUEDC2-silenced cells and BMP2 treatment (Fig. 5a, c), suggesting CUEDC2 specifically regulates the stability of SOCS3 in osteoblasts. Since CUEDC2 is involved in ubiquitin-mediated protein degradation^5^, we next determined whether CUEDC2 binds to SOCS3 and affects the ubiquitination of SOCS3. The binding between CUEDC2 and SOCS3 was confirmed using the co-immunoprecipitation assay (Fig. 5d). Moreover, we found that ubiquitinated SOCS3 proteins are reduced in CUEDC2-overexpressed cells (Fig. 5e), implying that CUEDC2 regulates SOCS3 protein stability by ubiquitin-mediated degradation.

To further investigate whether CUEDC2-mediated STAT3 phosphorylation is involved in osteoblast differentiation, we examined the effect of Stattic (a STAT3 phosphorylation inhibitor) on the osteogenesis of CUEDC2-silenced cells. Stattic was used at a 2.5 or 5 µM concentration, which is noncytotoxic but can inhibit phosphorylation of STAT3 (Fig. S1f, g). When CUEDC2 was silenced, the levels of osteogenic marker genes (ALP and BSP), ALP activity, and mineralized nodule formation were increased (Fig. 5f, g). Conversely, treatment with Stattic significantly inhibited Ad-shCUEDC2-induced osteoblast differentiation in a dose-dependent manner (Fig. 5f, g). These results suggest that CUEDC2 inhibits STAT3-mediated osteogenesis by regulating SOCS3 protein stability.

### CUEDC2 does not affect RANKL-induced osteoclast differentiation

**Fig. 6:**
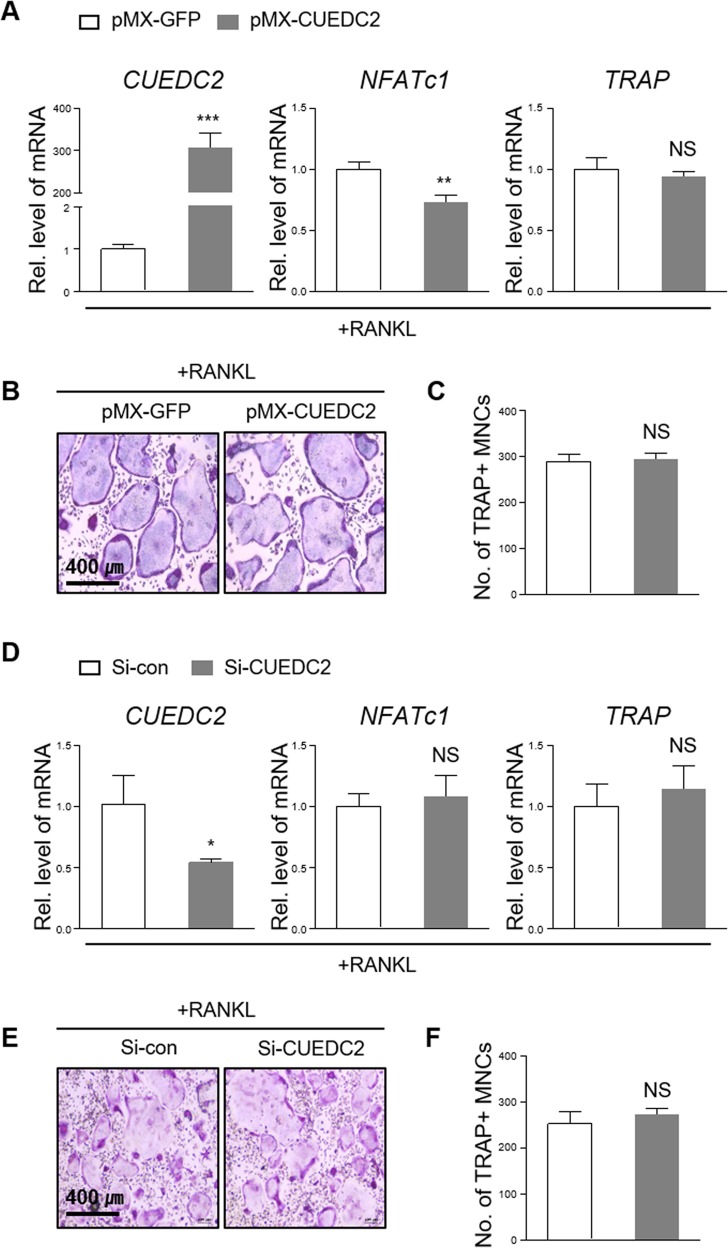
Effects of CUEDC2 on osteoclast differentiation. **a**–**c** Bone marrow macrophages were infected with pMX-IRES-EGFP (pMX-GFP) or CUEDC2 retrovirus, and the cells were then cultured with M-CSF (30 ng/ml) and RANKL (100 ng/ml) for 3 days. **a** Total RNA was extracted from the cultured cells, and then real-time PCR was performed using CUEDC2-, NFATc1-, and TRAP-specific primers. **P < 0.01, ***P < 0.001 versus pMX-GFP. **b** The cells were stained for TRAP. **c** The number of TRAP-positive multinucleated cells (MNCs) per well was counted. **d**–**f** BMMs were transfected with siRNA control or siRNA CUEDC2 (25 nM). After 24 h, cells were cultured for 3 days in the presence of M-CSF (30 ng/ml) and RANKL (100 ng/ml). **d** Real-time PCR analysis. *P < 0.05 versus si-control. **e** TRAP staining. **f** The number of TRAP-positive multinucleated cells.

In addition to osteoblasts, normal bone homeostasis is also controlled by osteoclasts. Therefore, the effects of CUEDC2 were also examined on osteoclast differentiation using gain- or loss-of-function experiments. When CUEDC2 was retrovirally overexpressed in BMM cells, the levels of NFATc1 mRNA, the master regulator of osteoclast differentiation, were significantly decreased (Fig. 6a). However, overexpression of CUEDC2 did not affect the levels of TRAP mRNA (Fig. 6a) and number of TRAP-positive multinucleated osteoclasts (Fig. 6b, c). These results were also observed in BMM cells where Cuecd2 was suppressed by si-CUEDC2 (Fig. 6d–f). Taken together, these data indicate that CUEDC2 does not affect osteoclast differentiation.

## Discussion

Here, we suggest for the first time that CUEDC2 is a negative regulator of osteogenesis. CUEDC2 mRNA and protein levels decreased during osteoblast differentiation. The overexpression of CUEDC2 attenuated BMP2-induced osteoblast differentiation, whereas the knockdown of CUEDC2 significantly enhanced osteoblast differentiation. Modulation of CUEDC2 levels in an in vivo bone-forming model resulted in either improved (knockdown) or regressive (overexpression) bone parameters in mice. CUEDC2 was found to inhibit osteoblast differentiation through the suppression of STAT3 phosphorylation by enhancing SOCS3 protein stability in preosteoblasts and BMSCs.

In the present study, we found that level of CUEDC2 is decreased by BMP2. On a related note, we attempted to elucidate how CUEDC2 expression is downregulated during osteoblast differentiation. A previous study showed that the CUEDC2 protein is upregulated during macrophage differentiation but decreased by interleukin-4 and miRNA 324-5p^28^. Interestingly, the expression of miRNA 324-5p in MC3T3-E1 cells was significantly increased by BMP2 (Fig. S3). However, it has been previously demonstrated that the overexpression of miRNA 324-5p inhibited osteoblast differentiation^29^. In this study, we did not investigate whether miRNA 324-5p modulation affects CUEDC2 expression in preosteoblasts or whether BMP2-induced miRNA 324-5p expression has a negative effect on osteoblast differentiation, as reported by the Young group^29^. Therefore, further investigation of these issues is needed.

We showed that CUEDC2 controls STAT3-mediated osteoblast differentiation by regulating SOCS3 protein stability. However, SOCS3 has been reported to inhibit osteoblast differentiation by directly regulating the phosphorylation of SMAD1, as well as STAT3^30^. In our study, phosphorylation and translocation of SMAD1 were unchanged by overexpression or knockdown of CUEDC2 (Fig. S4). Although further study is needed, we insist that CUEDC2 acts as a switch for osteoblast differentiation independently of SMAD1 by regulating the phosphorylation of STAT3 through interaction with SOCS3.

The activation of STAT3 directly induces the expression of factors related to the differentiation of osteoblasts and adipocytes^18,19,31^. Given our finding that CUEDC2 inhibits STAT3 activation, we investigated whether CUEDC2 could affect adipocyte differentiation of mesenchymal stem cells. Contrary to our expectations, the overexpression or knockdown of CUEDC2 did not affect adipocyte differentiation (Fig. S5). Although adipocyte differentiation is regulated by STAT3 activation, these results predict that the effect of CUEDC2 is probably by the indirect regulation of STAT3 activation via SOCS3. Studies have shown that suppression of SOCS3 expression affects insulin signaling but not adipocyte differentiation^32,33^. However, we did not investigate the levels of CUEDC2, SOCS3, and phosphorylated STAT3 during adipocyte differentiation. Therefore, further study is needed to determine whether CUEDC2-regulated SOCS3 protein is present upstream of STAT3 in adipocyte differentiation.

Recently, CUEDC2 is known to control the polarization of M1 macrophages by modulating the LPS- and Interferon-γ-induced NF-κB signaling pathway^28^. M1 macrophages have been proposed to be preosteoclasts that can be differentiated into osteoclasts by continuous RANKL exposure^34,35^. However, our study showed that CUEDC2 did not affect RANKL-induced osteoclast marker gene expression or TRAP-positive multinucleated cell numbers (Fig. 6). We assume that even though the polarization of M1 macrophages is facilitated by CUEDC2, the effects of CUEDC2 on osteoclast differentiation might be dependent on cell type-specific and ligand-specific activation of intracellular binding proteins. According to previous studies, Myd88 is an essential receptor-binding protein for LPS- or IL-1α-induced osteoclast differentiation, but this factor is not required for RANKL-induced osteoclast differentiation^36,37^. Meanwhile, in BMM, CUEDC2 was shown to regulate ligand (LPS, TNF-alpha, or IL-1α)-induced NF-κB activation, as well as the transcriptional activity of NF-κB induced by the overexpression of Myd88^6,28^. Therefore, further study is needed to determine whether CUEDC2 affects osteoclast differentiation under pathogenic conditions treated with LPS, TNF-alpha, or IL-1α, because CUEDC2 may specifically act on Myd88-dependent NF-κB signaling in BMMs.

In conclusion, this study suggests that CUEDC2 is a key regulator for osteoblast differentiation and bone formation via the regulation of STAT3 activation through interaction with SOCS3. Our findings highlight the possibility that CUEDC2 could be a new target for the treatment of bone regeneration and bone diseases.

## Supplementary information

Supplementary table

Supplementary Figure Legends

Supplementary figure 1

Supplementary figure 2

Supplementary figure 3

Supplementary figure 4

Supplementary figure 5
