# Supplementary material for: CUEDC2 controls osteoblast differentiation and bone formation via SOCS3–STAT3 pathway
Source: Cell Death Dis. 2020 May 11;11(5):344. doi: 10.1038/s41419-020-2562-5 (PMC7214468; doi:10.1038/s41419-020-2562-5)
Supplement: Supplementary file 1 — Supplementary table [file 41419_2020_2562_MOESM1_ESM.docx]

| Genes | Nucleotide sequence |
| --- | --- |
| *CUEDC2* | (F) 5′-AAGGATGACTTGAAGTCTTT-3′  (R) 5′-TCTCAGTGGAAGCGGTACTT-3′ |
| *ALP* | (F) 5′-TGAGGAAGAAGCCCATTCAC-3′  (R) 5′-ACTTCTTCTCCCGGGTGTG-3′ |
| *BSP* | (F) 5′-TTTCCCGTTCACCGTCCAC-3′  (R) 5′-ATCTTTGGTCTGGCTCCCATG-3′ |
| *OC* | (F) 5′-CTCCTGAGAGTCTGACAAAGCCTT-3′  (R) 5′-GCTGTGACATCCATTACTTGC-3′ |
| *NFATc1* | (F) 5′-CTCGAAAGACAGCACTGGAGCAT-3′  (R) 5′-CGGCTGCCTTCCGTCTCATAG-3′ |
| *TRAP* | (F) 5′-TCCGTGCTCGGCGATGGACCAGA-3′  (R) 5′-CTGGAGTGCACGATGCCAGCGACA-3′ |
| *PPARγ2* | (F) 5′-TCGCTGATGCACTGCCTATG-3′  (R) 5′-GAGAGGTCCACAGAGCTGATT-3′ |
| *ADIPOQ* | (F) 5′-CCTGGAGAAGCCGCTTATGT-3′  (R) 5′-CCTGGAGAAGCCGCTTATGT-3′ |
| *β-ACTIN* | (F) 5′-TTCTTTGCAGCTCCTTCGTTGCCG-3′  (R) 5′-TGGATGGCTACGTACATGGCTGGG-3′ |

Supplementary Table 1.

Table 1. Primers used for real-time PCR (mouse genes)
