# Supplementary material for: CUEDC2 controls osteoblast differentiation and bone formation via SOCS3–STAT3 pathway
Source: Cell Death Dis. 2020 May 11;11(5):344. doi: 10.1038/s41419-020-2562-5 (PMC7214468; doi:10.1038/s41419-020-2562-5)
Supplement: Supplementary file 2 — Supplementary Figure Legends [file 41419_2020_2562_MOESM2_ESM.docx]

**Supplementary Figures Legends**

**Fig. S1 Functional working concentrations of plasmid, adenovirus, and inhibitor were verified. a-e** overexpression or knockdown of CUEDC2 protein was evaluated by Western blotting analysis or immunohistochemistry (IHC). **a, c** MC3T3-E1 cells were transfected with Myc, Flag-CUEDC2 (200 ng), or si-CUEDC2 (25 nM). **b, d** Adenovirus (Ad-GFP or Ad-CUEDC2 or Ad-shCUEDC2) was added to MC3T3-E1 cells under GM conditions. **e** To confirm the overexpression of CUEDC2 in an ectopic mouse model, the samples from Fig. 3d were observed CUEDC2 by IHC. The arrowhead indicates positive staining of the CUEDC2 protein in the implant. **f, g** MC3T3-E1 cells were cultured with the indicated concentrations of Stattic. **f** Cell viability was evaluated using an MTT assay. ****P* < 0.001 versus Stattic 0 μM. **g** To determine the concentration of the STAT3 inhibitor, the phosphorylation of STAT3 was assessed by Western blotting.

**Fig. S2 CUEDC2 regulates osteoblast marker gene expression and mineralization in BMSCs.** **a-d** BMSCs were infected with Ad-CUEDC2 (50 MOI) or Ad-shCUEDC2 (50 MOI) for 4 h, and then infected cells were cultured with GM or OM. **a, b** Real-time PCR analysis. ***P* < 0.01, ****P* < 0.001 versus control of GM group. ^#^*P* < 0.05, ^##^*P* < 0.01, ^###^*P* < 0.001 versus the OM control group. **c** AR staining. **d** The intensity of AR staining. ****P* < 0.001 versus Ad-GFP with GM. ^#^*P* < 0.05, ^##^*P* < 0.01 versus Ad-GFP with OM.

**Fig. S3 MiRNA 324-5p expression increases during osteoblast differentiation.** MC3T3-E1 cells were cultured for 6 days under the conditions shown in Fig. 2a. For the quantification of miRNA 324-5p expression, cDNA synthesis was performed using an NCode VILO kit (Invitrogen). Real-time PCR was performed with 324-5p specific miRNA primers. Quantitation of 324-5p miRNA was normalized to sno234 RNA as an endogenous control. ***P* < 0.01 versus day 0.

**Fig. S4 CUEDC2 does not affect SMAD1 phosphorylation.** **a, b** MC3T3-E1 cells were infected with Ad-GFP (50 MOI), Ad-CUEDC2 (50 MOI), or Ad-shCUEDC2 (50 MOI). After infection, cells were cultured with BMP2 (50 ng/ml) for the time indicated in the figure. The cultured cells were subjected to the extraction of cytosol and nuclear protein (**a**) or whole protein (**b**) for Western blotting.

**Fig. S5 Effects of CUEDC2 on adipocyte differentiation. a-c** BMSCs were infected with pMX-IRES-EGFP (pMX-GFP) or CUEDC2 retrovirus, and the cells were then cultured with adipogenic medium [AM; Insulin (10 µg/ml), dexamethasone (1 µM), and rosiglitazone (2 µM)] for 5 days. **a** Total RNA was extracted from the cultured cells, and then real-time PCR was performed using specific primers. ****P* < 0.001 versus pMX-GFP. **b** The cells were stained for lipid droplets using oil red O stain solution. **c** For quantitative analysis, stained cells were incubated with isopropanol to extract the dye. The absorbance was measured at a wavelength of 510 nm on a spectrophotometer (Thermo Fisher Scientific). **d-f** BMSCs were transfected with Si-RNA (Si-con or Si-CUEDC2), and the cells were then cultured with AM for 5 days. **d** Real-time PCR analysis. **P* < 0.05 versus Si-con. **e** Oil red O staining. **f** Intensity of oil red O staining. NS, non-significant.
