# Supplementary figures and images for: CUEDC2 controls osteoblast differentiation and bone formation via SOCS3–STAT3 pathway
Source: Cell Death Dis. 2020 May 11;11(5):344. doi: 10.1038/s41419-020-2562-5 (PMC7214468; doi:10.1038/s41419-020-2562-5)

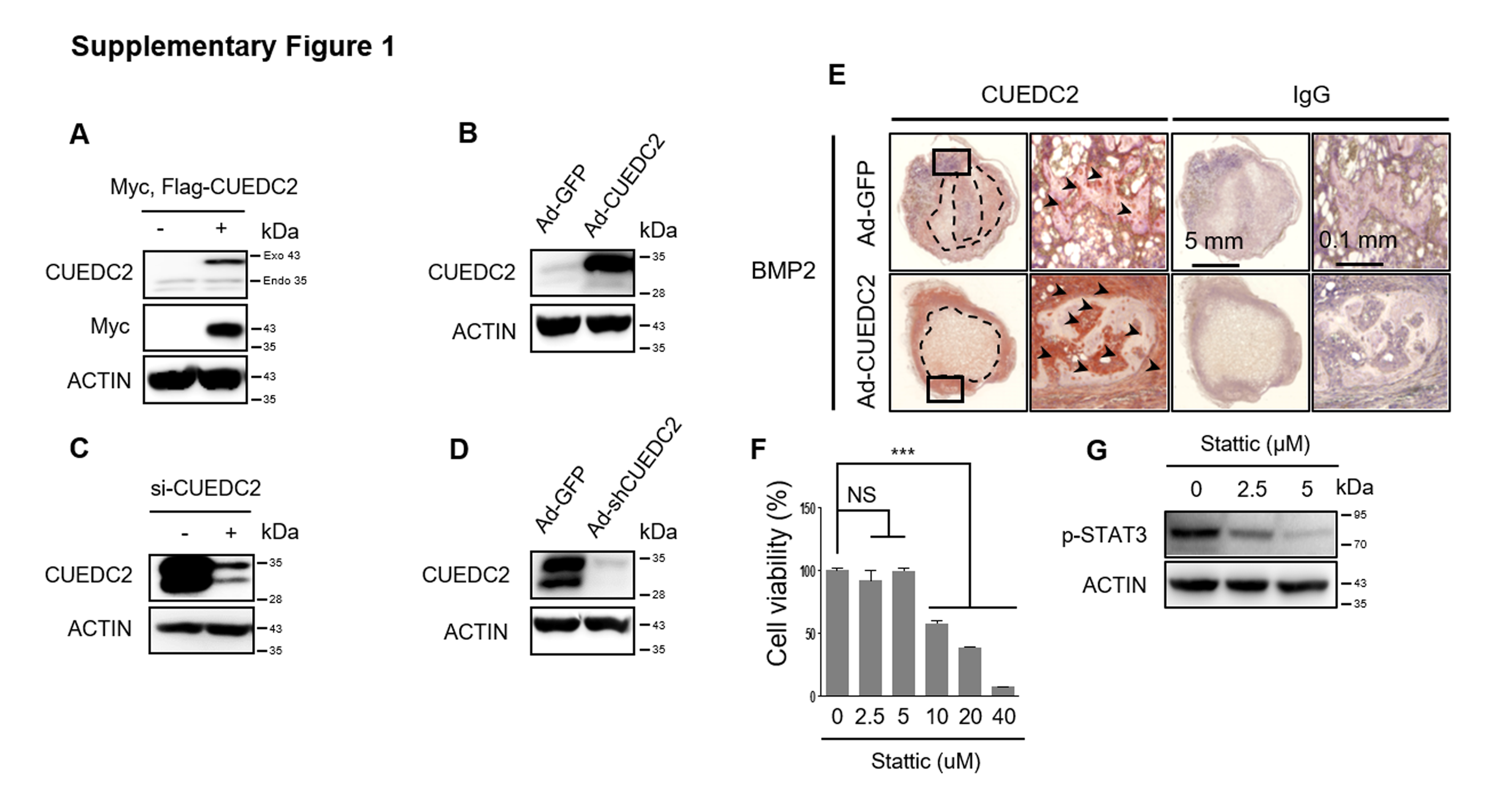

Supplement: Supplementary file 3 — Supplementary figure 1 [file 41419_2020_2562_MOESM3_ESM.tif]

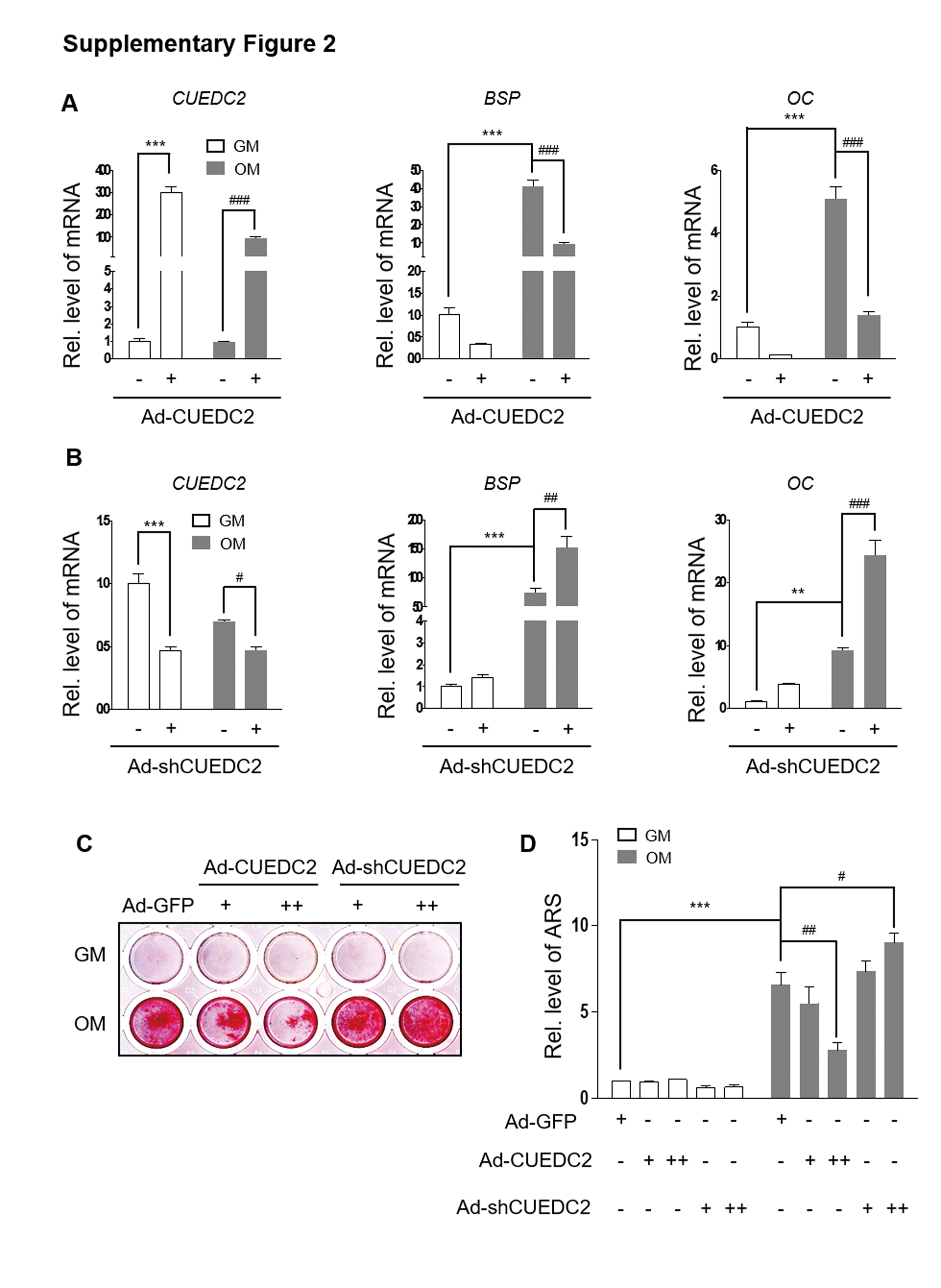

Supplement: Supplementary file 4 — Supplementary figure 2 [file 41419_2020_2562_MOESM4_ESM.tif]

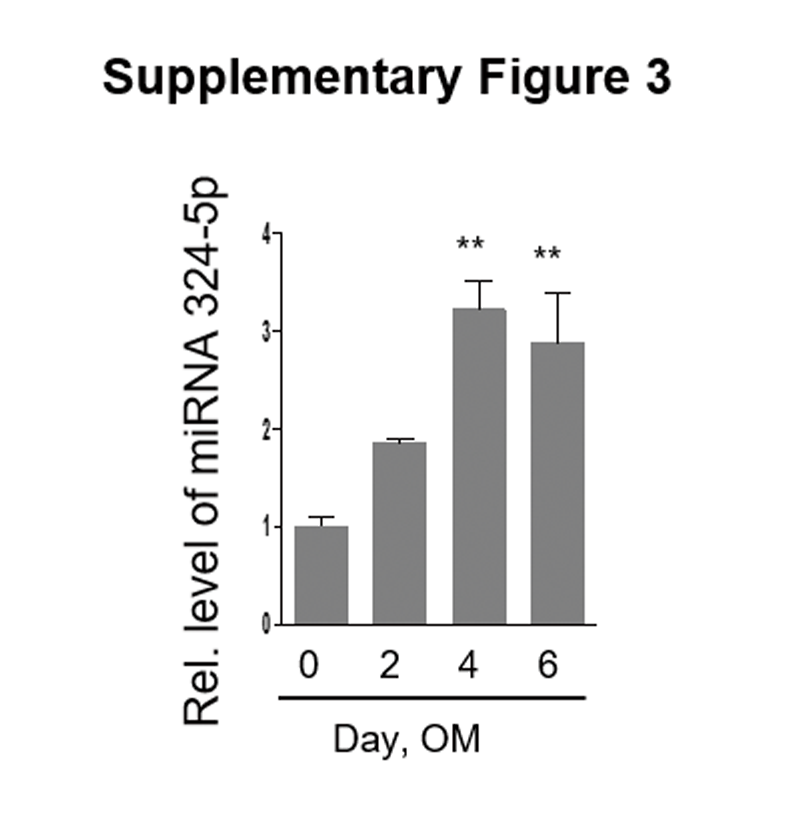

Supplement: Supplementary file 5 — Supplementary figure 3 [file 41419_2020_2562_MOESM5_ESM.tif]

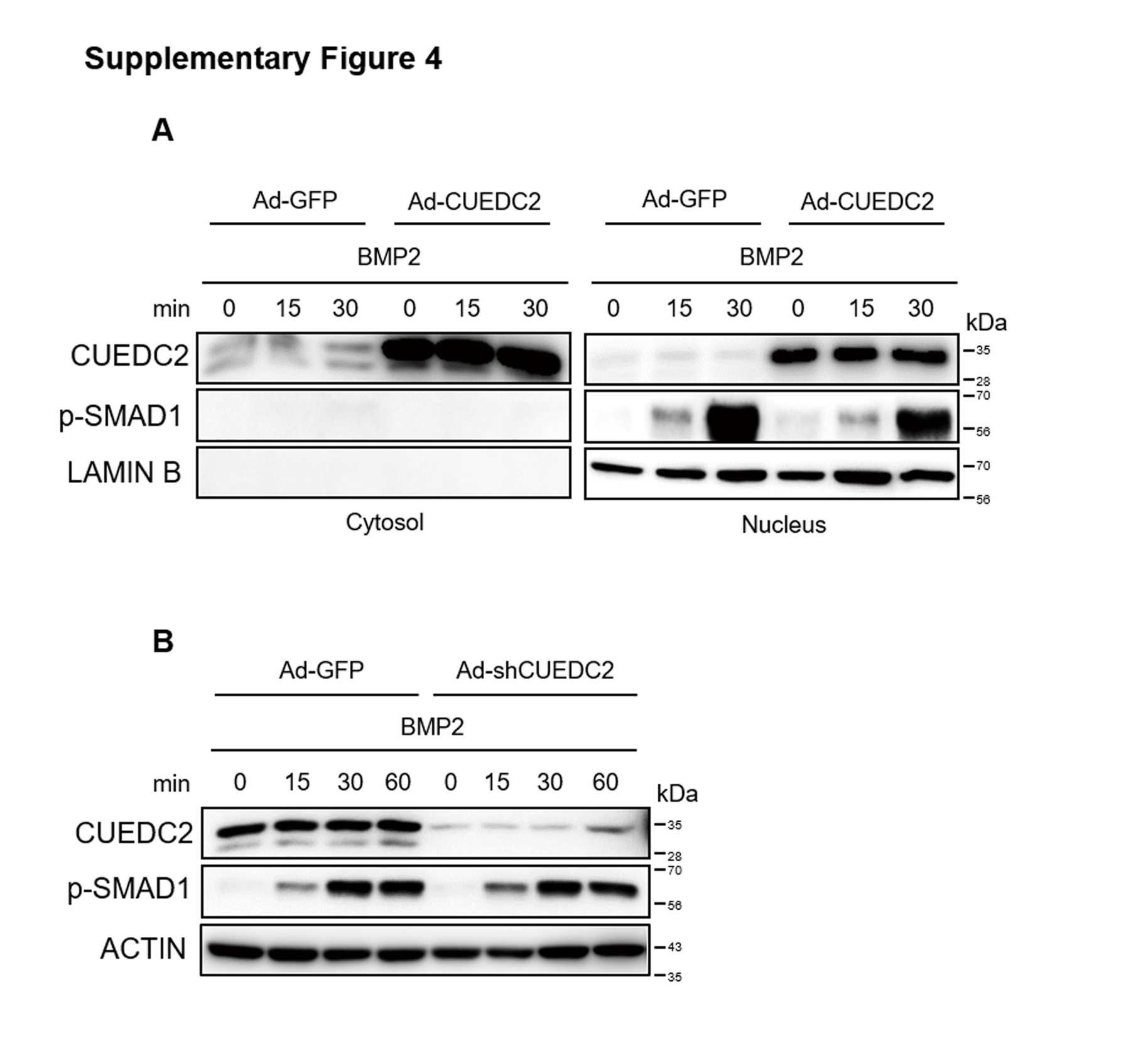

Supplement: Supplementary file 6 — Supplementary figure 4 [file 41419_2020_2562_MOESM6_ESM.tif]

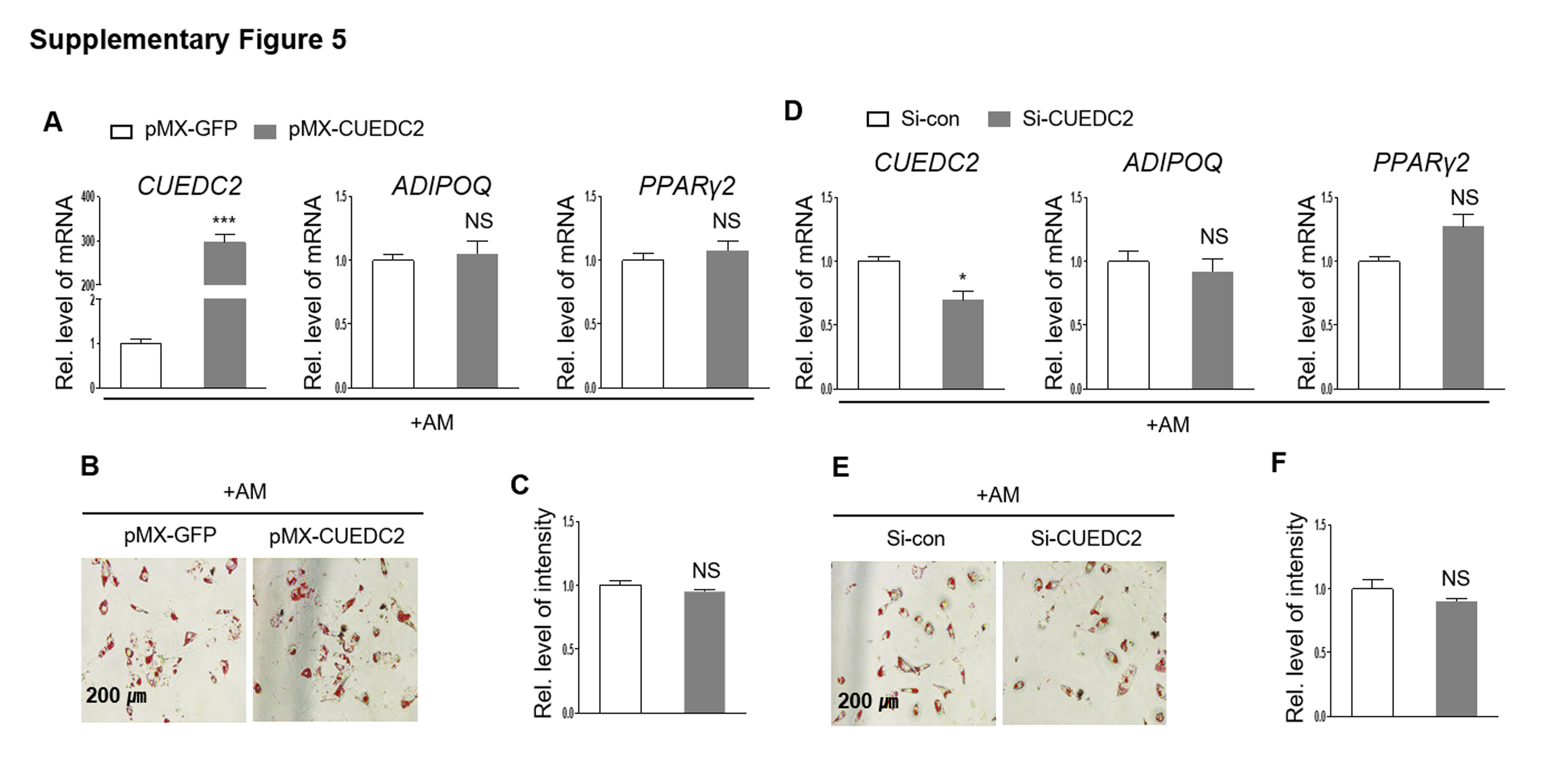

Supplement: Supplementary file 7 — Supplementary figure 5 [file 41419_2020_2562_MOESM7_ESM.tif]
